# Exceptional Evolutionary Divergence of Human Muscle and Brain Metabolomes Parallels Human Cognitive and Physical Uniqueness

**DOI:** 10.1371/journal.pbio.1001871

**Published:** 2014-05-27

**Authors:** Katarzyna Bozek, Yuning Wei, Zheng Yan, Xiling Liu, Jieyi Xiong, Masahiro Sugimoto, Masaru Tomita, Svante Pääbo, Raik Pieszek, Chet C. Sherwood, Patrick R. Hof, John J. Ely, Dirk Steinhauser, Lothar Willmitzer, Jens Bangsbo, Ola Hansson, Josep Call, Patrick Giavalisco, Philipp Khaitovich

**Affiliations:** 1 CAS Key Laboratory of Computational Biology, CAS-MPG Partner Institute for Computational Biology, Shanghai, China; 2 Max Planck Institute for Evolutionary Anthropology, Leipzig, Germany; 3 Graduate School of Chinese Academy of Sciences, Beijing, China; 4 Institute for Advanced Biosciences, Keio University, Tsuruoka, Yamagata, Japan; 5 Department of Anthropology, The George Washington University, Washington DC, United States of America; 6 Fishberg Department of Neuroscience and Friedman Brain Institute, Icahn School of Medicine at Mount Sinai, New York, United States of America; 7 Alamogordo Primate Facility, Holloman AFB, Alamogordo, New Mexico, United States of America; 8 Max Planck Institute for Molecular Plant Physiology, Potsdam, Germany; 9 Department of Exercise and Sport Sciences, Section of Human Physiology, University of Copenhagen, Copenhagen, Denmark; 10 Department of Clinical Sciences, Lund University, Malmö University Hospital, Malmö, Sweden; Massey University, New Zealand

## Abstract

Metabolite concentrations reflect the physiological states of tissues and cells. However, the role of metabolic changes in species evolution is currently unknown. Here, we present a study of metabolome evolution conducted in three brain regions and two non-neural tissues from humans, chimpanzees, macaque monkeys, and mice based on over 10,000 hydrophilic compounds. While chimpanzee, macaque, and mouse metabolomes diverge following the genetic distances among species, we detect remarkable acceleration of metabolome evolution in human prefrontal cortex and skeletal muscle affecting neural and energy metabolism pathways. These metabolic changes could not be attributed to environmental conditions and were confirmed against the expression of their corresponding enzymes. We further conducted muscle strength tests in humans, chimpanzees, and macaques. The results suggest that, while humans are characterized by superior cognition, their muscular performance might be markedly inferior to that of chimpanzees and macaque monkeys.

## Abstract 2

Accelerated evolution of the human brain and muscle metabolomes reflects our unique cognitive and physical capacities.

## Author Summary

Physiological processes that maintain our tissues' functionality involve the generation of multiple products and intermediates known as metabolites—small molecules with a weight of less than 1,500 Daltons. Changes in concentrations of these metabolites are thought to be closely related to changes in phenotype. Here, we assessed concentrations of more than 10,000 metabolites in three brain regions and two non-neural tissues (skeletal muscle and kidney) of humans, chimpanzees, macaque monkeys, and mice using mass spectrometry-based approaches. We found that the evolution of the metabolome largely reflects genetic divergence between species and is not greatly affected by environmental factors. In the human lineage, however, we observed an exceptional acceleration of metabolome evolution in the prefrontal cortical region of the brain and in skeletal muscle. Based on additional behavioral tests, we further show that metabolic changes in human muscle seem to be paralleled by a drastic reduction in muscle strength. The observed rapid metabolic changes in brain and muscle, together with the unique human cognitive skills and low muscle performance, might reflect parallel mechanisms in human evolution.

## Introduction

Metabolites, small molecules with molecular weight typically less than 1,500 Daltons, encompass a broad range of compounds that includes building blocks of proteins, nucleic acids, and lipid membranes, energy and chemical group carriers, and neurotransmitters and other signaling molecules. Changes in metabolite concentrations closely reflect changes in the physiological states of tissues and cells. Thus, knowledge of metabolite concentration changes specific to human tissues may be instrumental to understanding the molecular mechanisms underlying evolution of the human phenotype. Genomic and gene expression surveys have demonstrated how comparative studies of humans and closely related primate species can lead to identification of evolutionarily, physiologically, and medically relevant features characteristic of humans [1],[2], including changes related to metabolism [3]. Indeed, several physiological features specific to humans imply possible metabolic adaptations. For instance, evolution of a large brain in humans, which consumes 20% of the total body energy at rest, has been suggested to involve changes in energy distribution among tissues, as well as adaptation to a nutrition-rich diet [4],[5]. Similarly, emergence of endurance running in hominid ancestry [6] may have added another constraint to human metabolic evolution.

Despite the potential significance of metabolic changes to evolution of the human phenotype, few studies of human-specific metabolic features exist. Previous studies, although limited to 21 and 118 metabolites measured in brains of humans, chimpanzees, and macaque monkeys, revealed extensive metabolite concentration differences among species affecting approximately 50% of the assessed compounds [7],[8]. To further deepen our understanding of metabolite concentration changes accompanying recent human evolution, as well as to shed light on the general dynamics of metabolome evolution, we conducted a comprehensive metabolome survey across five tissues of humans, chimpanzees, rhesus monkeys, and laboratory mice based on over 10,000 metabolic compounds. In each species, we examined metabolite concentrations in three brain regions, prefrontal cortex (PFC), primary visual cortex (V1), and cerebellar cortex (CBC), as well as in the kidney cortex and thigh skeletal muscle of 14 adult healthy individuals (Table S1). Small molecules present in the tissue samples were assayed using the following methodologies: (*i*) gas chromatography-mass spectrometry (GC-MS), (*ii*) positive mode liquid chromatography-mass spectrometry ([+]LC-MS), and (*iii*) negative mode liquid chromatography-mass spectrometry ([−]LC-MS). The effects of postmortem delay, as well as environmental effects characteristic of the industrialized human lifestyle—a diet rich in fat and sugar content, low levels of exercise, and high levels of social stress—were assessed using the same set of metabolite measurements in 14 additional macaque monkeys (Table S2). In total, metabolite measurements were performed in 365 tissue samples. To test the reliability of metabolite measurements further, a subset of samples from four individuals per species per tissue was measured using capillary electrophoresis-mass spectrometry (CE-MS) as an alternative procedure. Metabolic measurements were accompanied by the measurements of the expression of enzymes using RNA-sequencing (RNA-seq) in a subset of 120 tissue samples.

## Results

### Metabolome Quantification

**Figure 1:**
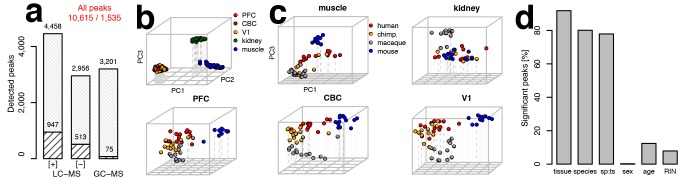
Metabolome data quantification, annotation, and variation analysis. (a) The total number of metabolite peaks measured in our datasets. The bottom part of each bar indicates the number of peaks that were annotated in respective datasets. The total numbers of peaks and numbers of annotated peaks are indicated above the bars. (b) PCA plot based on normalized intensities of 10,615 peaks from all metabolic datasets measured in five tissues of the four species. Each circle represents an individual sample colored according to the tissue. (c) PCA plots for each of the five tissues: muscle, kidney, PFC, CBC and V1. Each circle represents an individual sample colored according to species. (d) Percentages of peaks with a significant proportion of variation (ANOVA, permutation *p*<0.01) explained by tissue, species, species–tissue interaction term (sp∶ts), sex, age, and sample RNA preservation. RIN, RNA Integrity Number.

Across these datasets, we quantified a total of 10,615 peaks (mass spectrometry features), which were detected in at least one tissue of one species (Figure 1a). Among them, 1,535 could be annotated using metabolite standards or computationally matching the metabolite features to metabolite database entries. As all human and most chimpanzee samples were not collected immediately after death, we removed from further analysis 636 peaks with concentrations affected by postmortem delay (Table S3). These peaks were identified in comparison between macaque samples collected with (*n* = 2) and without (*n* = 17) postmortem delay (*q*<0.01 in at least one tissue, Figure S1). Out of 371 metabolites detected in the independent CE-MS experiment, 265 were detected by the LC-MS and GC-MS methodologies. The concentration profiles of these metabolites across the different species showed significant agreement among methodologies in all tested tissues (Wilcoxon test, *p*<0.001, Figure S2).

Examination of metabolite concentration variation by means of principal component analysis (PCA) showed a clear separation of samples of all four species according to the three major organs—brain, kidney, and muscle—in each dataset (Figure 1b, Figure S3). Within each tissue we could further observe grouping according to species identity (Figure 1c). Accordingly, of the 10,615 detected metabolite peaks, 92% showed significant concentration differences among tissues (permutation *p*<0.01, false discovery rate (FDR)<1%), using analysis of variance (ANOVA). Further, 80% of peaks showed significant concentration differences among the four species and 61% among primates (permutation *p*<0.01, FDR<2%), indicating large scope and potential functional importance of metabolome divergence among species. By contrast, the effects of sex, age, and tissue preservation on metabolite concentration variation were substantially smaller: on average <1, 12, and 8% of all the assayed metabolite peaks were affected by one of these factors, respectively (Figure 1d, Table S4).

### Metabolome Differences among Tissues

**Figure 2:**
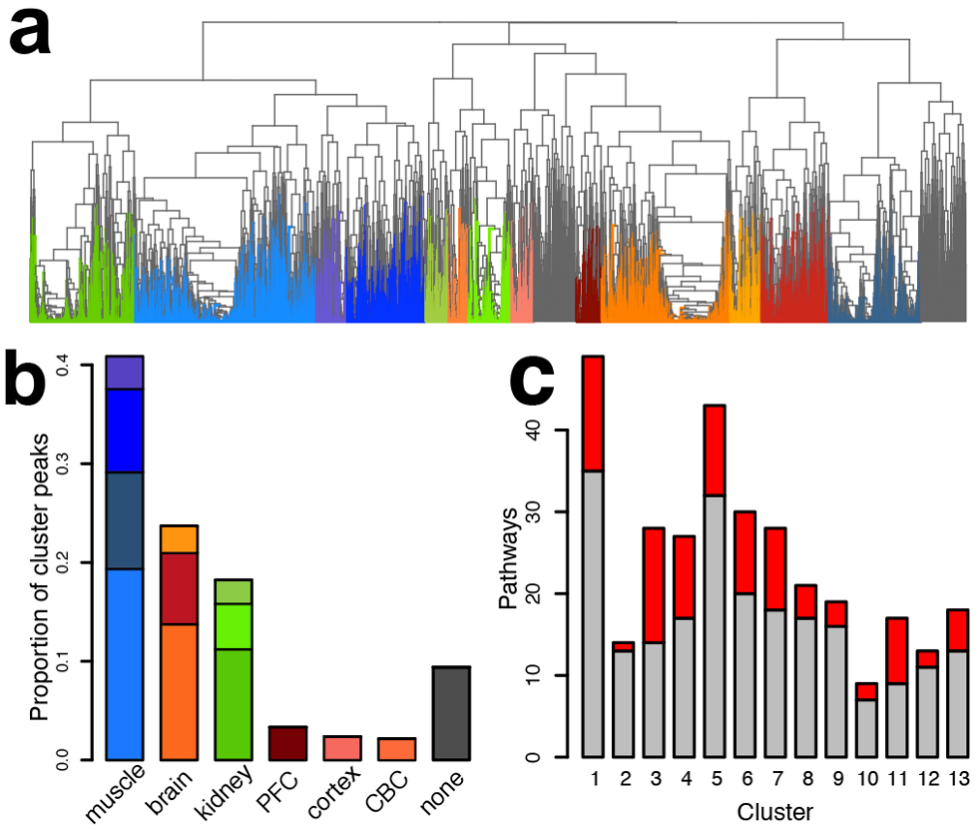
Tissue-specific metabolite clusters. (a) Dendrogram of 9,603 metabolite peaks after hierarchical clustering, based on concentration profile correlations across tissues in all four species. The colors represent 13 metabolite clusters colored according to the tissue-specificity of the cluster concentration profiles: shades of red indicating brain-specific clusters, shades of blue: muscle-specific, and shades of green: kidney-specific clusters. Clusters cumulatively containing less than 10% of all assayed peaks were removed from further analyses and are colored in gray. (b) Proportions of peaks specific to a given tissue based on the cluster analysis. Colors of the bars correspond to the colors of clusters in panel a. (c) Numbers of pathways with significant enrichment of metabolites in the 13 metabolite clusters. The red part of each bar shows pathways with significant overrepresentation of enzymes with matching tissue-specific expression profiles.

To examine the metabolic differences among tissues further, we sorted 9,603 metabolite peaks that showed significant concentration differences among the tissues into 13 metabolite clusters using nonsupervised hierarchical clustering (Figure 2a, Figure S4). Most of the clusters contained metabolite concentration profiles specific to a given tissue (Figure S5). The largest proportion of metabolite concentration profiles was muscle-specific (41%), almost 2-fold more than brain-specific (23%) and kidney-specific (18%).

All clusters contained metabolic changes enriched in specific pathways (permutation *p*<0.01), many of them associated with known tissue-specific functions (Table S5). To test whether the metabolite concentration differences among tissues could be confirmed against differences in the expression of their corresponding enzymes, we measured transcript levels in 120 of the 365 tissue samples using RNA-seq (Tables S1, S6). In this dataset we detected expression of 17,913 protein-coding genes that could be mapped among primate species (16,398 between primates and mouse), with 3,820 (3,720) of them linked to metabolites based on the Human Metabolome Database Version 3.0 (HMDB) annotation (Figure S6). By combining metabolite concentration and gene expression measurements, we showed that pathways containing excess of tissue-specific metabolic profiles also showed significant over-representation of corresponding tissue-specific gene expression profiles in all 13 metabolite clusters (permutation *p*<0.05, Figure 2c). Specifically, clusters containing metabolites and enzymes showing brain-specific profiles were enriched for, among others, neuroactive ligand-receptor interaction and the synaptic vesicle cycle; muscle-specific profiles for glycolysis/gluconeogenesis and oxidative phosphorylation; and kidney-specific profiles for aldosterone-regulated water reabsorption (Table S5). Thus, metabolite concentration differences among tissues could be confirmed by expression-level differences in corresponding genes, and these metabolite concentration differences represent some of the known functional properties of the tissues.

### Metabolome Divergence among Species

**Figure 3:**
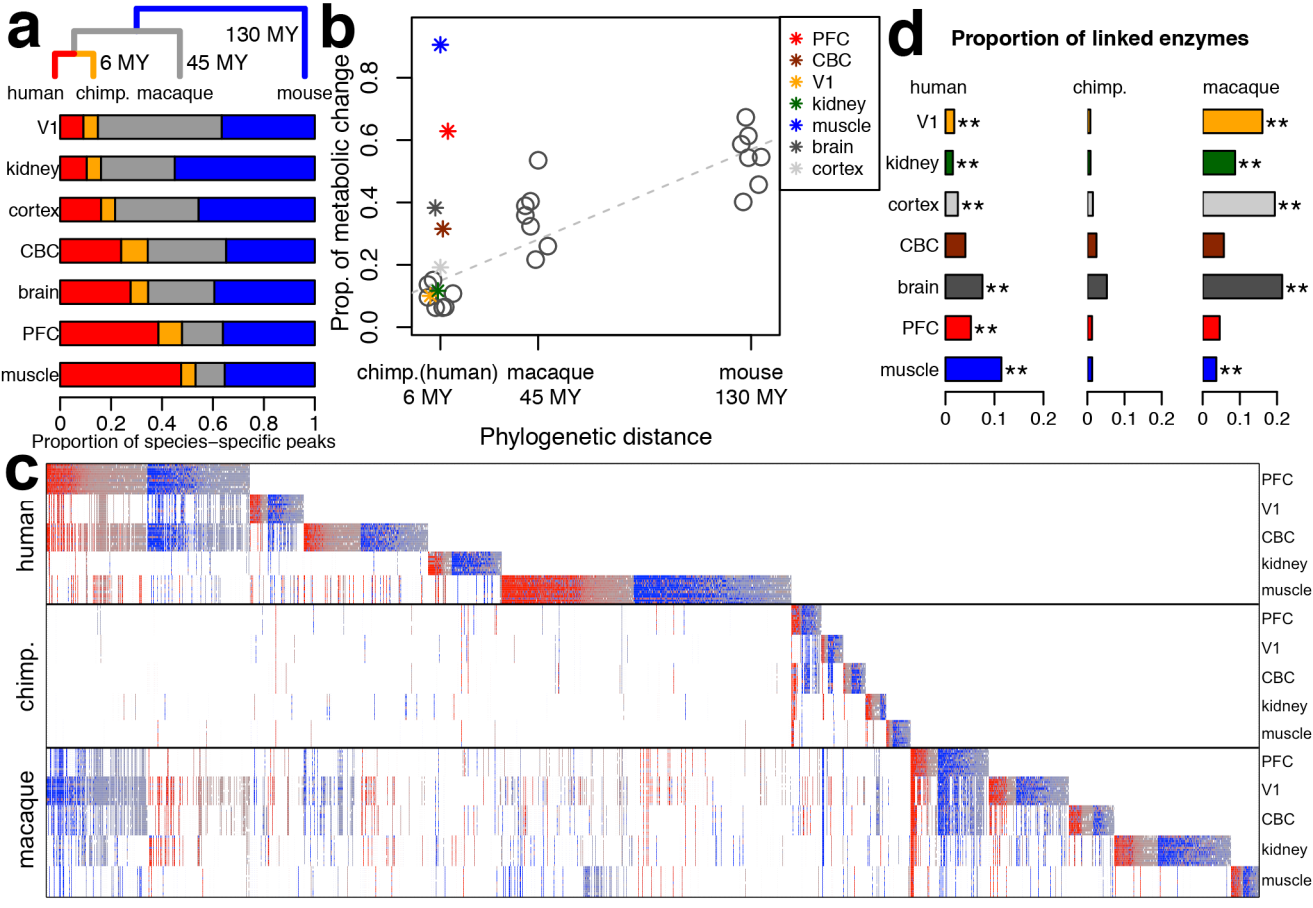
Species-specific metabolic change. (a) Proportions of metabolite concentration changes on the four species evolutionary lineages. The phylogenetic tree above the bars shows the human (red) and chimpanzee (orange) evolutionary lineages, the lineage connecting the common ancestor of humans and chimpanzees with macaques (gray), and the lineage connecting the common ancestor of humans, chimpanzees, and macaques with mice (blue). The colors within the bars show proportions of metabolite peaks with concentration changes specific to the corresponding lineages. (b) Relationship between species-specific metabolite concentration divergence in each tissue and the corresponding phylogenetic distances in million years (MY). The linear regression between metabolite concentration divergence and phylogenetic distances represented by the dashed line was calculated based on the proportions of metabolite peaks with significant concentration divergence on the corresponding lineage for the three nonhuman species (represented by circles). Asterisks colored according to the tissues show the proportions of metabolite peaks with significant concentration divergence on the human lineage relative to the chimpanzee linage. (c) Heat map of relative metabolite concentration levels across three primate species. The plot is based on the 4,698 metabolite peaks that showed significant concentration differences in any tissue in the three primate species (*t*-test, permutation *p*<0.01). The columns represent metabolite peaks; the rows represent 190 tissue samples sorted by species and tissue identity. For each peak, concentration level in a given sample was normalized to the concentration across all samples of other primate species within the same tissue (base 2 logarithm-transformed ratios). The colors show positive (red) and negative (blue) concentration differences from the mean expression level. Concentration levels, *p*-values and annotation of the peaks shown in the Figure are listed in Table S7. (d) Proportions of enzymes with matching species-specific expression profiles linked to species-specific metabolites in a given tissue normalized by the number of enzymes with expression profiles specific to any species in this tissue. Tissues showing significant overrepresentation of enzymes with matching species-specific expression profiles are indicated with asterisks: **, *p*<0.01 and *, *p*<0.05.

To inspect metabolic divergence among species, we sorted 9,356 metabolite peaks that showed significant concentration differences among the four species (ANOVA, species or tissue–species terms, permutation *p*<0.01, FDR<2%) into species-specific categories within each of the five tissues, as well as within composite tissue categories: cerebral cortex (PFC+V1) and brain (PFC+V1+CBC). The greater number of samples belonging to cerebral cortex and brain, compared with individual tissue categories, results in the greater statistical power of tests performed on these composite tissue samples. For this reason, throughout the study we compared the relative proportions of change among species within each tissue, without comparing the differences in species-specific change among composite and individual tissues. As a result, we found that in all tissues metabolic divergence on the chimpanzee, macaque, and mouse evolutionary lineages could to a large extent be explained by genetic distances among these species [9] (Pearson *r^2^* = 0.88, *n* = 21, *p*<0.01). By contrast, the extent of metabolic divergence on the human evolutionary lineage differed substantially among tissues (Figure 3a,b; Figure S7). Human-specific metabolic divergence in the V1 cortical region and kidney was similar to chimpanzee-specific divergence in these tissues and could be predicted from regression fit of metabolic divergence based on phylogenetic divergence times of the three nonhuman species (Figure 3a). By contrast, in the brain, PFC, and skeletal muscle, human-specific metabolic divergence exceeded chimpanzee-specific divergence by 4.0-, 4.2-, and 8.4-fold respectively. This excess was significantly greater than expected from phylogenetic time-based divergence estimates (linear regression *p*<0.01). In fact, human-specific metabolic divergence in the sampled thigh skeletal muscle (mainly vastus lateralis) and PFC exceeded average metabolic divergence seen in the mouse tissues. This indicates that during the last 6–7 million years of human evolution, human muscle metabolome underwent greater change than the mouse metabolome did over the approximately 130 million years separating mice from the common ancestor of humans, chimpanzees, and macaques.

The remarkable excess of human-specific metabolic divergence in the skeletal muscle, PFC, and in the brain as a whole, but not in V1 or the kidney, could be clearly visualized when grouping all species-specific metabolic changes found on the human, chimpanzee, and macaque evolutionary lineages (Figure 3c, Table S7). This result was also observed for the three mass spectrometry datasets analyzed separately, was robust at various statistical significance cutoffs, could be validated by metabolite measurements overlapping between LC- and GC-MS techniques, was valid for both annotated and unannotated metabolite peaks, and was not affected by exclusion of metabolites with low concentration levels (Tables S8, S9, S10, Figures S8, S9). More importantly, human-specific metabolic changes showed significant agreement with expression profiles of the linked metabolic enzymes in all tissues except one (CBC, Figure 3d). Similarly, significant agreement between metabolite concentration and enzyme expression profiles was seen on the macaque evolutionary lineage for tissues containing large numbers of macaque-specific metabolic changes: V1, kidney, cerebral cortex, whole brain, and skeletal muscle.

All groups of metabolites showing species-specific concentration levels in different tissues were significantly enriched in metabolic pathways from Kyoto Encyclopedia of Genes and Genomes (KEGG) database resource (permutation *p*<0.01, Table S11). Of the 115 KEGG pathways containing an excess of species-specific metabolic changes, 100 contained at least one enzyme showing a consistent species-specific expression pattern in the RNA-seq data. Furthermore, in 61 of the 100 pathways, the metabolites and enzymes that showed consistent species-specific profiles were directly linked, while only 12 pathways would be expected by chance (permutation *p*<0.01). At the individual pathway level, 44 pathways contained significant excess of enzymes with matching species-specific expression profiles either directly or indirectly linked to the corresponding species-specific metabolites (permutation *p*<0.01, Table S11) while only seven pathways would be expected by chance (permutation *p*<0.01).

**Figure 4:**
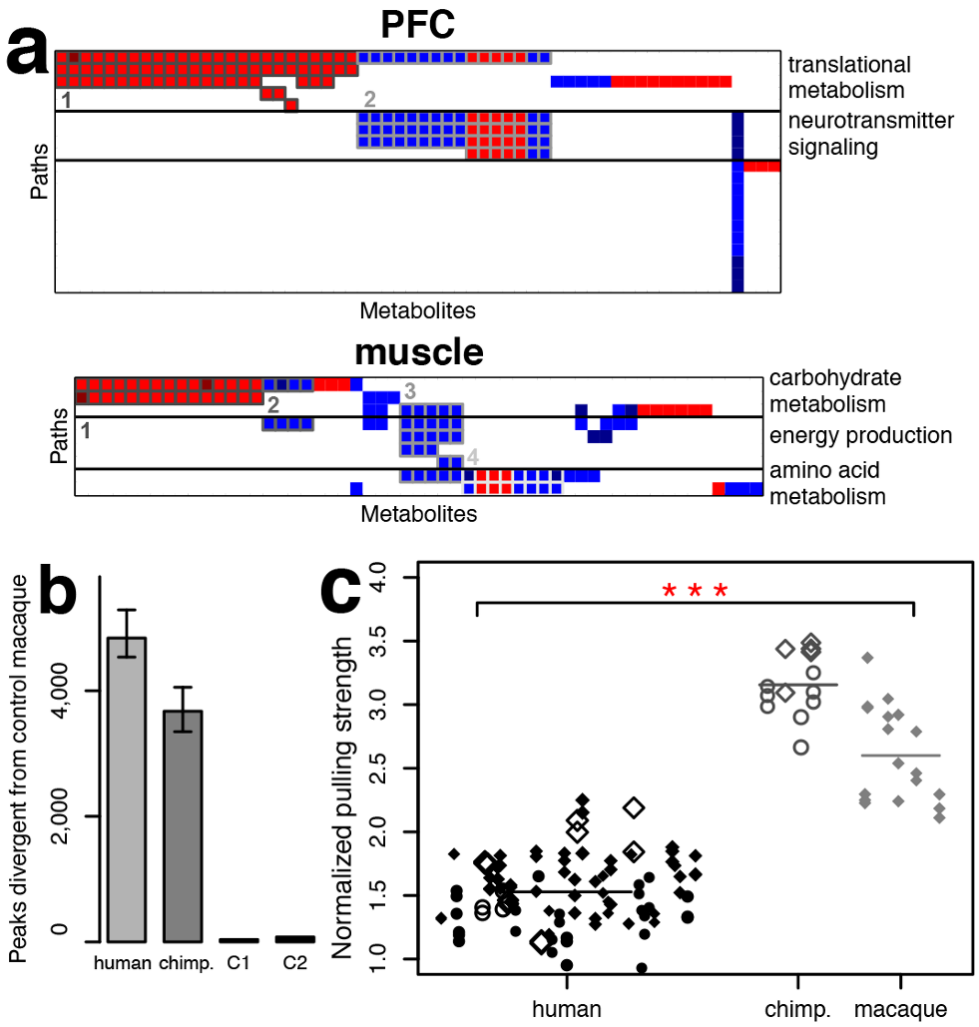
Functions of the human-specific metabolites, environmental effects, and muscle strength experiments. (a) Functional units of KEGG pathways containing significant excesses of metabolites with human-specific concentration profiles in PFC and skeletal muscle. Columns represent metabolites showing human-specific concentration profiles in these tissues. Rows represent KEGG pathways enriched in the respective metabolites. Colors show higher (red) and lower (blue) concentration levels in humans compared with the other two primate species. Only pathways supported by human-specific expression of enzymes are shown. Metabolites directly linked to the enzymes with a human-specific expression profile in these tissues are indicated by darker shades of red and blue. Numbers and borderlines of different shades of gray indicate metabolite groups showing correlated concentration profiles across multiple pathways. Plotted metabolites, pathways, and metabolite groups are listed in Tables S14, S15 and further illustrated in Figures S10, S11. (b) Numbers of metabolite peaks differentiating humans, chimpanzees, and macaques subjected to environmental condition 1 (C1: physical activity deprivation), and macaques subjected to environmental condition 2 (C2: high nutrition and physical activity deprivation) from the control macaques in any of the five tissues (*t*-test, *p*<0.01). Numbers of peaks showing significant concentration differences between control macaques and humans or chimpanzees were estimated using six randomly chosen human or chimpanzee individuals. The error bars show 0.05 and 0.95 quintiles of the significant peak number distributions calculated by randomly sampling six human or six chimpanzee individuals 1,000 times. (c) Pulling strength of humans, chimpanzees, and macaques normalized by individual body weight. The shape of the symbols represents the individual's sex: circles: females, diamonds: males. Empty symbols represent trials measured using the apparatus built in the Leipzig primate center, filled symbols those measured using the apparatus built in Shanghai. The size of the symbols is proportional to the individual's age normalized by the maximal life expectancy of the species: humans: 120 years, chimpanzees: 60 years, macaques: 40 years. Up to three of the best results per individual are shown. Asterisks indicate the significance of muscle strength difference between humans and the other two species (Wilcoxon test, *p*<0.001). The highest measurement of each individual was used in the *p*-value calculation.

Among the 47 pathways that contained an excess of human-specific metabolic changes supported by enzyme expression, 20 were found in the PFC and 9 in skeletal muscle (Tables S12, S13). Based on shared metabolites, these pathways could be further grouped into functional units. Human-specific metabolic changes in the PFC included two such units: *(i)* translational metabolism (valine, leucine, and isoleucine degradation, selenocompound metabolism, aminoacyl-tRNA biosynthesis); and *(ii)* neurotransmitter signaling (dopaminergic synapse, cocaine and amphetamine addiction) (Figure 4a, Table S14, Figure S10). In muscle, functional units partially overlapped and reflected human-specific changes in: *(i)* carbohydrate metabolism (propanoate metabolism, pentose and glucuronate interconversions, glycolysis/glucogenesis); *(ii)* energy production (oxidative phosphorylation); and *(iii)* amino acid metabolism (histidine, β-alanine metabolism) (Figure 4a, Table S15, Figure S11). Additional human-specific metabolic changes representing separate functional units were also observed in other tissues (Tables S16, S17, S18, Figure S12).

### Extent of Environmental Effects on Metabolome Composition

Could the stark excess of human-specific metabolic changes found in some of the examined tissues be caused by environmental factors, such as diet, stress, or physical activity? To address this, we performed metabolome measurements in 12 additional macaque monkeys (male, 2–14 years old) that were subjected to conditions designed to reproduce some aspects of the modern human lifestyle. Specifically, the test macaques were placed indoors in solitary standard size cages with no dietary change (stress and exercise factors: condition 1, *n* = 6) or with an additional dietary change to a cooked diet with high sugar and fat content (stress, exercise, and diet factors: condition 2, *n* = 6) for a 1-month period (Table S2). Similar dietary changes introduced over a shorter time interval in mice were previously shown to reproduce some of the gene expression differences observed between human and chimpanzee livers [10]. Prior to this treatment, the test macaques, as well as all other macaques used in this study (standard condition: controls, *n* = 17), were fed a balanced plant-based diet and housed in family groups in spacious outdoor enclosures. Environmental differences between test macaques and macaques kept at standard conditions had only a modest effect on metabolite concentrations: a total of 37 and 78 metabolic peaks were affected in at least one of the five tissues by condition 1 and condition 2, respectively (permutation *p*<0.01, Figure 4b). By contrast, an average of 3,674 and 4,840 metabolic peaks showed equally significant differences in comparisons between control macaques and six randomly selected chimpanzee or human individuals, respectively (Figure 4b, Figure S13). The metabolic changes that were induced by environmental effects showed significant enrichment in 19 KEGG pathways (permutation *p*<0.01, Table S19) containing a total number of human-specific metabolic change (68 peaks) significantly higher than expected in randomly sampled pathways (permutation *p*<0.05). The significant overlap between environmentally induced and human-specific metabolic changes indicates that, despite the limited scope and duration of the environmental change, it nevertheless did capture some of metabolic effects of the modern human lifestyle. The extent of metabolic changes induced by environmental perturbation was, however, limited and constituted less than 3% of all human-specific metabolic changes identified in our study.

### Muscular Strength Differences among Primates

The relatively small effect of the short-term environmental changes on metabolite concentrations in macaque tissues indicates that the extraordinary human-specific metabolic divergence observed in skeletal muscle, PFC, and brain might reflect the phenotypic differences between humans and other species. We hypothesize that rapid metabolic evolution in brain and skeletal muscle is associated with unique cognitive and physical abilities of humans. While cognitive functions clearly distinguish humans from other primates, to our knowledge only anecdotal observations exist suggesting that human muscular strength could be inferior to that of other primates [11]–[15]. To investigate this, we conducted a series of experiments aimed at providing an approximate estimate of muscular strength in a pulling task accomplished by humans (*n* = 42), chimpanzees (*n* = 5), and macaques (*n* = 6) (Movie S1). The task consisted of pulling suspended adjustable weights in a setup involving both upper and lower parts of the body and conducted in a procedure allowing us to estimate the maximum pulling strength of an individual. In this test humans showed substantially inferior performance compared with chimpanzees and macaques, with an average strength difference of almost 2-fold (Wilcoxon test, *p*<0.001, Figure 4c, Table S20). Prolonged training is known to increase human strength [16], therefore the tested humans included 16 individuals engaged in regular physical activities, among them five university team basketball players and four professional climbers. By contrast, all tested nonhuman primates were raised in captivity and were never subjected to physical training. Many additional factors including ones potentially favoring nonhuman primates, such as difference in muscle mass distribution between front and hind limbs [17],[18], as well as ones favoring humans, such as motivation to perform, could not be accounted for in our experiment. The inability to control nonhuman primates' motivation hinders the exact quantification of the muscular strength difference between humans and nonhuman primates, even in a highly controlled experimental setup. Nonetheless, our results indicate the existence of systematic differences in muscle performance between humans and nonhuman primates.

## Discussion

Our study provides several novel insights into the nature and the potential mechanisms of metabolic evolution. First, we demonstrate that while metabolic divergence among mammalian species tends to reflect genetic distances among species, stark exceptions to this rule exist. Specifically, we show that the metabolomes of PFC and skeletal muscle have undergone 4- and 7-fold greater divergence, respectively, on the human evolutionary lineage than expected from genetic divergence. Notably, this observation contrasts with evolutionary divergence at the transcriptome level where overall transcriptome divergence largely follows genetic distances in all tissues (Figure S14).

Second, we show that metabolite concentrations are largely buffered against environmental changes, such as temporary change in diet, physical activity, and living conditions. While this is true for tissues assessed in our study—brain regions, kidney, and muscle—this observation might not apply to tissues closely interacting with the environment, such as liver and blood. Moreover, exposure to altered environmental conditions over a longer time span could result in more pronounced changes. Nevertheless, the 2 months' exposure of macaques to “human-like” environmental conditions did recapitulate some human-specific metabolic changes, indicating the relevance of our environmental tests.

The third and perhaps most unexpected observation is a striking excess of human-specific metabolic divergence in skeletal muscle. While excess of human-specific metabolic divergence in brain or brain regions such as PFC might relate to cognitive functions unique to humans, the functional significance of extreme metabolic divergence of human skeletal muscle is less apparent. Our results suggesting a major difference in muscular strength between humans and nonhuman primates provide one possible explanation for this phenomenon. In humans, muscle tissue is known to have variable fiber-type composition, reflecting differences in individuals' physiology and physical activity [19]. Since different muscle fiber types have different functional and metabolic properties [20], systematic difference in fiber-type composition between human and other primate species could explain our observations of extensive metabolic differences and reduced human muscular strength. To assess this hypothesis, we measured fiber-type composition in nine adult chimpanzee individuals (Table S21). Variation in fiber-type composition observed among chimpanzees was, however, indistinguishable from that observed among humans [19]. Thus, differences in fiber-type composition between humans and nonhuman primates are not likely to explain the extensive metabolic divergence, as well as difference in strength, observed in our study. Further experiments are needed to investigate the exact extent of muscle performance differences between humans and nonhuman primates, as well as the link between changes in muscle performance and muscle metabolite composition on the human lineage. On the molecular level, such experiments could involve isolation of individual muscle types or functional groups and testing of their contractile properties. On the organism level, more controlled experiments could inspect recruitment of particular muscles in motion and quantify their performance based on their volume and level of activation.

While the molecular mechanism linking metabolic divergence with changes in muscular strength on the human evolutionary lineage cannot be determined based on our observations alone, we hypothesize that metabolic evolution of human muscle and brain metabolomes may have occurred in parallel. Studies demonstrating a connection between aerobic exercise and cognitive performance in humans of different age indicate that these two organs might be metabolically related [21]. Furthermore, it was also previously suggested that rescaling of energetically expensive organs, such as the gut, allowed the development of a larger brain in human evolution [4]. Our results indicate that the reallocation of energy to energetically costly human brains may have required further decrease in energy expense in the skeletal muscle, at least during peak performance. Combined with previous observations of decreased bone robustness potentially reflecting a decline in muscular strength on the human evolutionary lineage [22], it is plausible that human brain and muscle were evolving in a reciprocal manner to adjust to the increasing energy demands of the growing brain [5] and to adapt to new types of physical activities requiring greater endurance, both characteristic of modern humans [6]. While differences in muscle energy consumption between humans and other primates are currently unknown, we speculate that metabolic coevolution of muscle and brain may require either an overall decrease in muscle energy consumption or, alternatively, a shift to alternative energy sources, such as lipids, thus realizing more glucose for an energy-demanding human brain. Accelerated changes in metabolite concentrations in brain and skeletal muscle on the human evolutionary lineage may, therefore, represent some of the molecular underpinnings of this evolutionary process.

## Materials and Methods

### Ethics Statement

The studies were reviewed and approved by the Institutional Animal Care and Use ethics committee at the Shanghai Institute for Biological Sciences. For all teenagers participating in this study, we obtained written consent from their parents in addition to the subjects' assent.

### Samples

Human samples were obtained from the National Institute of Child Health and Human Development (NICHD) Brain and Tissue Bank for Developmental Disorders at the University of Maryland, the Netherlands Brain Bank, China's Wuhan Xiehe Hospital, and the Chinese Brain Bank Center. Written consent for the use of human tissues for research was obtained from all donors or their next of kin. All subjects were defined as healthy controls by forensic pathologists at the corresponding tissue bank. All subjects suffered sudden death with no prolonged agonal state. Chimpanzee samples were obtained from the Alamogordo Primate Facility, New Mexico, USA, the Anthropological Institute & Museum of the University of Zürich-Irchel, Switzerland, and the Biomedical Primate Research Centre, the Netherlands. Rhesus macaque samples were obtained from the Suzhou Experimental Animal Center, China. All nonhuman primates used in this study suffered sudden deaths for reasons other than their participation in this study and without any relation to the tissue used. Mouse samples were obtained from the mouse facility at Shanghai Institute for Biological Sciences. All mouse individuals were from the C57/BL6 strain with no genetic modifications. Since mouse measurements were used solely to compare the pace of metabolic and genetic divergence among species, underestimation of metabolic and genetic variation within mouse species because of their genetic homogeneity does not affect the main results of our study.

The PFC samples were dissected from the anterior part of the superior frontal gyrus, a cortical region approximately corresponding to Brodmann area 10. The V1 samples were dissected from the calcarine cortex (human and chimpanzee) or the occipital lobe (macaques), corresponding to Brodmann area 17. The CBC samples were dissected from the lateral part of the cerebellar hemispheres. In all brain samples we took special care to dissect gray matter only. The kidney samples were dissected from the renal cortex. Muscle tissue was dissected from thigh skeletal muscle, mainly vastus lateralis. All tissues were snap-frozen after dissection and stored at −80°C without thawing.

Each tissue sample was of approximately 100 mg of weight and was extracted from the frozen postmortem tissue on dry ice without de-freezing. The tissue samples were first powdered using a mortar and pestle cooled by liquid nitrogen and then separated into two parts for the metabolite and RNA extraction procedures.

### Postmortem Samples

In order to identify metabolites affected by postmortem delay, we collected tissue samples from two macaque monkeys that were dissected and frozen 4–6 hours after death (postmortem samples). Other macaques used in this study had a postmortem interval of less than 15 minutes.

### Condition 1 and Condition 2 Treatments

To assess the environmental effects on the measured metabolites, we collected samples of two additional groups of macaques fed a calorie-rich diet and exposed to limited physical activity. Twelve male macaque monkeys were used for this purpose. All selected monkeys had no medical history of abnormal behavior and were healthy, based on biochemical and blood tests. The monkeys were randomly assigned to three groups, *control* (three individuals), *condition 1* (six individuals) and *condition 2* (six individuals). For a period of 4 weeks, monkeys from the control group continued with their routine lifestyle while monkeys from the other two groups were kept in special conditions.

Monkeys from the control group were maintained on a standard *ad libitum* feeding regimen, with two meals each day at 900 and 1600 hours, consisting of 1,100 calories (20 biscuits) of Purina High Protein Monkey Diet, jumbo size (W.F. Fisher and Son Inc.), and one-quarter piece of fresh fruit for the morning meal. These individuals were housed in spacious group cages hosting family groups of about 20 individuals and providing open and walled living spaces of about 120 m^3^ in total.

Macaques from condition 1 and condition 2 groups were transferred from the standard living conditions (control condition) into individual standard-size cages located in a temperature-controlled room (24±2°C) with lights on 12 hours each day (700–1900). The cage size was close to 1 m^3^ and largely limited their space for movement. The diet of condition 1 monkeys was kept unchanged and was the same as the diet of monkeys from the control group. The diet of condition 2 monkeys was based on cooked food, consisting of 100 g rice, one medium size egg, 50 g peanuts, 15 g sugar, and 100 g potato daily, which amounted to the total energy of approximately 1,060 calories. The animals accepted this type of food and the diet was maintained unchanged for 1 month until the animals were humanely killed.

The studies were reviewed and approved by the Institutional Animal Care and Use ethics committee of the Shanghai Institute for Biological Sciences.

### GC- and LC-MS Sample Preparation and Measurements

Metabolites were extracted from the frozen tissue powder by a methanol: methyl-tert-butyl-ether (1∶3 [v/v]) extraction according to Giavalisco et al. [23]. In brief, 50 mg of frozen powdered tissue material was re-suspended in 1 ml extraction solution containing 0.5 µg of corticosterone, 1.5 µg of 1,2-diheptadecanoyl-*sn*-glycero-3-phosphocholine (Avanti Polar Lipids, 850360P), 0.5 µg of 13C sorbitol and 0.25 µg of ampicillin. The samples were incubated for 10 min at 4°C on an orbital shaker. This step was followed by ultrasonication in a bath-type sonicator for 10 min at room temperature. Finally, the insoluble tissue material and the precipitated proteins were pelleted by a centrifugation step (5 min; 14,000 g) and the supernatant transferred to a fresh 2 ml Eppendorf tube. To separate the organic from the aqueous phase, 500 µl of an H_2_O∶methanol mix (3∶1 [v/v) was added to the supernatant, vortexed, and centrifuged (5 min; 14,000 g). Subsequently, 500 µl of the upper, organic phase, plus 650 µl of the polar phase (150 µl for GC-MS and 500 µl for LC-MS) were collected to three 1.5 ml Eppendorf tubes. Each aliquot was concentrated to complete dryness in a speed vacuum centrifuge at room temperature. Extract derivatization and GC-MS measurements were performed according to Lisec et al. [24].

In the LC-MS datasets, in addition to the individual tissue samples, we measured mixtures of tissue samples (pooled samples). Pooled samples were measured after every 40^th^ sample, providing us information on system performance (sensitivity and retention time consistency), sample reproducibility, and compound stability over the time of the MS-based analysis.

### CE-MS Sample Preparation and Measurements

Metabolites were extracted from the frozen tissue powder by 1 ml methanol containing 20 µM each of L-Methionine sulfone (WAKO: 502-76641), 2-Morpholinoethanesulfonic acid, monohydrate (DOJINDO 349-01623), and sodium d-camphor-10-sulfonic acid (WAKO: 037-01032). Then, 0.5 ml of lysate was transferred to an Eppendorf tube containing 500 µl chloroform and 200 µl water. After 30 seconds of vortexing and 15 min of centrifugation at 4°C, 300 µl, the aqueous phase was transferred to an ultrafiltration tube (Millipore). The filtered liquid was concentrated to complete dryness in a speed vacuum for 3 hours at 35°C.

The remaining 0.5 ml tissue lysate was incubated at room temperature for 30 min after adding 4 ml of chloroform, 1.4 ml methanol, and 0.1 ml 30 µM sphingomyelin (Avanti) in a 10 ml glass tube. After 10 min of centrifugation, the supernatant was collected and dried in a speed vacuum.

### RNA-seq Sample Preparation and Measurements

From a total set of tissue samples used in metabolite measurements, we selected, based on the RNA preservation, a subset of six samples from each species and each tissue (120 samples in total) for the analysis of gene expression. Total RNA was extracted by a TRIzol (Invitrogen) procedure according to the manufacturer's instructions. All RNA quality was assessed using an Agilent 2100 Bioanalyzer. Only samples with high RNA integrity values were used in this study.

Sequencing libraries were prepared using the TruSeq RNA-seq Sample Preparation Kit (Illumina) according to the manufacturer's instructions. Briefly, polyadenylated RNA was isolated using a poly-dT bead procedure and then chemically fragmented and randomly primed for reverse transcription. After second-strand synthesis, the ends of the double-stranded complementary DNA were repaired. After 3′-end adenylation of these products, Illumina Single-End Sequencing adapters were ligated to the blunt ends of the cDNA fragments. Ligated products were then PCR-amplified (15 cycles). The RNA-seq libraries were sequenced (100 cycles) on the Illumina HiSeq 2000 platform.

### Peak Alignment

What we define as a peak throughout this manuscript is an individual mass trace together with isotopes (mass spectroscopy feature) at a distinct retention time in the chromatographic separation (chromatographic feature). LC-MS peaks were aligned across samples according to Giavalisco et al. [23], using the Refiner MS software package version 6.0 (GeneData). GC-MS peaks were extracted and annotated using msPeak (Steinhauser et al., unpublished). In this procedure the GC-MS chromatograms were exported as NetCDF files (ANDI MS format) using Leco ChromaTOF software (version 3.25) with the following settings: baseline subtraction just above the noise (1.0), peak width broadening with width-to-time (w/t) of 3.5/0 seconds—6.0/1,400 seconds, and without smoothing. Peak smoothing (nine data points) and alignment were performed within msPeak for each mass trace separately using peak spread and peak gap to neighboring peaks.

To limit the potential technical artifacts, we employed a multistep peak filtering procedure to all MS datasets. The LC-MS datasets were filtered according to the following criteria:

1. retention time (RT) ≥0.6 min,
2. peaks present in ≥80% of pooled samples with an intensity of ≥1,000 arbitrary units (AU),
3. peaks showing consistent concentration levels in the first two pooled samples of the LC-MS datasets, which corresponds to the maximal time that any of the samples were kept in the autosampler before measuring,
4. peaks present at the level of ≥10,000 AU in ≥50% of samples of any particular species and tissue.

Before applying the last filtering criterion, outlier samples were removed from the datasets. Outliers were identified based on PCA plots and included nine samples in the [+]LC-MS and [−]LC-MS datasets. This filtering procedure resulted in 4,458 [+]LC-MS peaks and 2,956 [−]LC-MS peaks satisfying all the criteria, which corresponds to 6% of the initially registered peaks.

The GC dataset was filtered according to following criteria:

1. peaks distinct from the background, which we define as a measurable peak width at the level of 10% of its apex,
2. RT of a peak in all samples within the range of 2 min around the median RT of this peak,
3. RT outside of the range of 0.75 min of the RTs of any of the used internal standards (IS) RTs or, if the peak shares the same mass with any of the IS, outside of the range of 1.5 of the RT of this IS,
4. peak level ≥3,000 AU in ≥50% of samples of samples of any particular species and tissue,
5. peaks with the same mass and RT differing by ≤0.5 were classified as overlapping and removed.

Before applying criteria 4, nine outlier samples identified based on PCA analysis were removed from the dataset. The filtering resulted in 3,201 peaks in this dataset, representing 9% of the initial peak set.

In the quantitative part of our analysis, we used the entire set of unannotated peaks satisfying the above quality criteria. This set was narrowed down to the identified molecules in further functional analysis.

### Normalization

All peak concentration values were log transformed and quintile normalized. The normalization was performed on groups of samples belonging to the same tissue, an approach that was selected out of several other methods [25] according to normalization-quality measures of peak variation within samples of the same tissue and species.

### RNA-seq Reads Alignment and Mapping

We used Bowtie2 [26] with a default parameter setting to align the reads to the respective genomes: human version hg19, chimp panTro3, rhesus rheMac2, and mouse version mm9 from the Ensembl66 [27]. Bowtie2 reports up to two possible alignments for each read. For further analysis we kept reads with alignment score >150 and, among those that mapped to two locations, only those with a difference in alignment score ≥5. Among reads that mapped to an exon and exon junction, those with higher scores were kept. The one that mapped to an exon was kept if the scores were equal. Following this procedure, we obtained 24.1 M mapped reads per sample on average. Gene expression was quantified as (N*100)/(L-99) where N is the number of reads aligned to gene exons and L is the total length of the gene exons. We used the liftOver tool [28] to map the human genome onto the genomes of the other three species. Comparison of gene expression among three primate species was based on the genome mapping reported by the liftOver tool. For reconstruction of the mouse and human junctions, we additionally used mouse genome annotation and homology information. In the comparison of mouse to other species, we restricted the set of genes' exons to those that could be mapped among all four species. In the comparison of gene expression among primates, we used a larger set of genes' exons that could be mapped among the three primate species. As a cutoff for a minimal level of expression of the analyzed genes, we selected the 5% quintile of all gene nonzero expression levels in all samples.

### Hierarchical ANOVA

We used analysis of variance (ANOVA) to identify metabolite peaks showing significant concentration level differences among species or tissues, or significant species–tissue interaction term (F-test *p*<0.01). ANOVA was performed on all samples and on the samples of primates only (primate test). For peaks showing a significant proportion of variation explained by species and species–tissue terms in ANOVA, we performed an additional *t*-test to identify the species in which the metabolite peak concentration levels were significantly different from the other species. Metabolite peaks significant in the primate ANOVA test were tested for peak specificity to any primate species; peaks significant in the ANOVA test performed on all species samples were tested for mouse-specific concentration levels. In the *t*-test, samples from each tissue were tested separately. In each test, peak levels of a given species were compared with the peak levels in the samples of the remaining species—other primates if the tested species were primate, all primates if the tested species was mouse. ANOVA and *t*-test significance levels were corrected for multiple testing and the FDR levels at different nominal test significance levels were estimates by sample label permutations. Peaks showing *p*<0.01 and FDR<5% in 1,000 permutations were considered significant.

In addition to the initial set of five tissues that the samples were collected from, we defined two composite neural tissues obtained by *in silico* grouping of specific brain regions: cortex tissue was defined as a combination of two cortical regions: PFC and V1; brain tissue was defined as a combination of all three brain regions. In the *t*-test used to identify species-specific peak concentration levels in these tissues, all samples of the composite tissues were used.

In analyses of species-specific metabolic changes unique to the five individual tissues used in this study, we excluded metabolite peaks with concentration levels specific to a given species and showing the same direction (increase or decrease) of change in multiple tissues within three tissue groups: *(i)* PFC and V1; *(ii)* cortex and CBC; *(ii)* brain, kidney, and muscle.

### Identification of Environment- and Postmortem Delay-Induced Metabolite Concentration Changes

In order to identify metabolite peaks with concentration levels altered by changes in diet, stress, and physical activity in macaques, we performed a *t*-test on the tissue samples from condition 1 (*n* = 6) or condition 2 (*n* = 6) macaque individuals and the control tissue samples of all macaque individuals with unaltered environmental conditions (*n* = 17) in each tissue. Peaks showing significant difference (*t*-test *p*<0.01, permutation *p*<0.01) in condition 1 or condition 2 samples and showing the same direction of change in the human samples (elevated or decreased) were classified as affected by environmental change in condition 1 or condition 2, respectively. The overlap with human-specific metabolite concentration changes was determined as an overlap of environment-induced changes in a given tissue, and metabolite concentration differences showing the same direction (elevated or decreased) in this tissue in the human samples as compared with the control macaques.

In order to identify metabolite peaks with concentration levels altered by postmortem delay, we quantified the differences in peak concentration levels in each tissue between the macaque samples from individuals living in control conditions collected with 4- and 6-hour postmortem delay (*n* = 2) and macaque samples from individuals living in control conditions collected with less than 15 min postmortem delay (*n* = 17). Peaks showing concentration differences in the top or bottom 5% quintile of all peak differences between postmortem samples and control samples were excluded from further analyses in the respective tissue.

### Clustering of Metabolite Concentration Differences among Tissues

We performed hierarchical clustering with complete linkage of metabolite peaks. Silhouette value was used to choose the best clustering. Peaks in the smallest clusters, the total size of which were <10% of all peaks within a metabolite class, were excluded from further cluster analysis.

### Gene Expression Analysis

Based on the gene expression measurements of the 120 tissue samples we searched for genes showing expression profiles specific to a particular tissue and specific to a particular species in a given tissue. For each gene we calculated the mean difference of its expression level between every pair of tissues and between every pair of species in each tissue. Genes showing differences in expression level in a particular tissue compared with all other tissues, or species compared with all other species in the same tissue, within the upper or lower 5% quintile (10% quintile in the tissue comparison) of the difference distribution were classified as tissue-specific or species-specific in a given tissue respectively.

### Peak Annotation and Pathway Analysis

We used accurate mass searches with a mass tolerance of 5 ppm, allowing [M+H], [M+NH_4_] and [M+Na] as possible adducts in positive ionization mode samples and [M−H] and [M+Formic acid-H] in negative ionization mode samples [23]. The obtained m/z values were then searched against the HMDB database [29] for annotation of the [+]LC-MS and [−]LC-MS datasets. For the annotation of the GC-MS dataset, all of the GC-MS dataset chromatograms were processed and aligned as described previously [30],[31] using several reference compound libraries of commercially available authentic standards measured on the employed GC-MS system [31]–[33].

We used the resulting annotation to assign the metabolites to specific KEGG [34] pathways and inspected metabolic pathways overrepresented in the two types of metabolite groups found in our analysis: clusters of all peaks and peaks showing species-specific concentration levels in different tissues. We used metabolite annotation from both HMDB and KEGG databases to link the metabolites to metabolic pathways. Hypergeometric test (*p*<0.05, permutation *p*<0.05) was used to identify pathways with significant overrepresentation of metabolites belonging to a specific metabolite group, as compared with other metabolites measured in our study. In the search for KEGG pathways with significant overrepresentation of metabolites showing species-specific concentration profiles, we used all metabolites measured in this study as a background for the hypergeometric and permutation tests. In the search for KEGG pathways with significant overrepresentation of metabolites from 13 metabolite clusters, we used all metabolites belonging to clusters with concentration profiles specific to tissues different from the tissue of the tested cluster as a background for the hypergeometric and permutation tests.

### Agreement between Metabolite and Gene Expression Data

We assessed the agreement between metabolite concentration levels and expression of their linked enzymes. Among genes detected in the transcriptome data, we extracted those reported by the KEGG database as enzymes linked, based on reaction information, to the metabolites measured in our metabolite datasets. Next, we tested the overrepresentation of enzymes with expression profiles matching concentration profiles of metabolites among: *(i)* enzymes annotated in pathways with significant overrepresentation of metabolites showing species-specific concentration profiles in a tissue, as well as pathways with significant overrepresentation of metabolites from tissue-specific metabolite clusters; *(ii)* enzymes directly linked to metabolites based on KEGG annotation in the tissue-specific clusters or the species-specific metabolites.

In the pathway-based analysis *(i)* we tested both the overrepresentation of pathway enzymes with a corresponding expression profile, as well as pathway enzymes with a corresponding expression profile directly linked to the pathway metabolites with species-specific concentration profile or belonging to a tissue-specific cluster. In the link-based analysis *(ii)* we tested only the overrepresentation of enzymes showing corresponding expression profiles directly linked to the cluster metabolites or species-specific metabolites in KEGG annotation. In this test, in an iterative procedure, we randomly sampled sets of metabolites of the same size as the tested metabolite group—species-specific or tissue-specific cluster metabolites—and calculated the proportion of enzymes of the matching expression profile among all enzymes connected to the sampled metabolite. In the random permutations, we only used metabolites reported to interact with any of the enzymes measured in our transcriptome analysis. In the analysis of clusters and cluster pathways, the metabolites were sampled from all metabolites belonging to clusters with concentration profiles specific to a tissue different from the tissue of the tested cluster. The proportion of enzymes with the corresponding expression profile linked to the tested metabolite group—pathway *(i)* or cluster *(ii)*—was compared with the proportion obtained in the sampling test.

### Metabolite and Pathway Functional Groups

To facilitate functional analysis of metabolites showing species-specific concentration profiles in different tissues and KEGG pathways with significant overrepresentation of these metabolites, we grouped pathways according to shared metabolites into functional units. Following the pathway enrichment analysis, in each species-specific metabolite group, metabolites belonging to two or more KEGG pathways and sharing more than two-thirds of the overrepresented pathways were incrementally grouped into functional units until no two metabolites sharing sufficient numbers of pathways could be grouped together. This process resulted in functional units and metabolite groups representing different metabolic functionality.

### Fiber Typing of Chimpanzee Muscle Samples

The frozen biopsy samples were placed in a 9 mg/ml sodium chloride solution and stored at 5°C for 30 min [35]. Afterwards excess moisture was thoroughly removed from the samples with absorbent paper towels before the samples were mounted in an embedding medium (Tissue-Tek OCT Compound, Sakura Finetek) and frozen in isopentane precooled with liquid nitrogen. Four serial 10 mm-thick sections were cut at −30°C, preincubated at pH 4.37, 4.6, and 10.3 and stained for myofibrillar ATPase reactions at pH 9.4 [36]. Subsequently, fibers were classified under light microscopy as type I, IIa, and IIx fibers.

### Muscle Strength Measurements

We designed an experimental setup for testing muscular strength of humans, chimpanzees, and macaques that would be applicable to all three species to a similar extent. Specifically, the measurements were conducted using a simple mechanical apparatus consisting of a sliding shelf attached to a handle on one side, and suspended adjustable weights on the other side of the shelf. In chimpanzee and macaque experiments, the handle was placed inside a cage and the individual was encouraged to displace the shelf (by pulling from the handle) to bring food within her reach. Pulling on the handle required the individual to lift the respective weight attached to the shelf by engaging both upper and lower body parts. The strength of the pull was registered by an electronic scale placed on the shelf and attached to a rope connecting the handle and the weight. In order to register the maximal pulling force, the electronic scale was continuously filmed by a camera placed on the moving shelf. With each individual's successful pull, the amount of weight attached to the device was increased. After several failed attempts by an individual to pull a certain weight, the amount of food on the device was increased. The maximum pull force measured by the electronic scale divided by the individual's body weight was used as a normalized measure of an individual's strength.

For nonhuman primate experiments, six healthy male macaques of 5–6 years of age with body mass 7–10 kg and five chimpanzees (three females and two males) of 8–11 and 37 years of age and body mass 32–55 kg were tested. All selected individuals had no medical history or reported abnormal behavior and were healthy during the strength experiments. Macaques were borrowed from breeding facilities at Suzhou Experimental Animal Center, China, where the tests were performed, and returned to the breeding facility after the tests. Macaque monkeys, prior to the muscle tests, were housed in spacious enclosures of approximately 120 m^3^. Each enclosure hosted a group of approximately 20 individuals and contained multiple objects used for climbing. Chimpanzee tests were performed at the Wolfgang Köhler Primate Research Center of the Max Planck Institute for Evolutionary Anthropology in Leipzig Zoo, Germany. The Zoo enclosures are large and contain objects imitating a natural chimpanzee environment, including climbing facilities.

For human experiments, a total of 42 human individuals were tested. Thirty-six of them were tested using the Suzhou apparatus and eight using the Leipzig apparatus. Human individuals included 29 adults 17–34 years of age with a wide span of body mass between 42 and 127 kg (mean 64 kg±15 kg SD). Among these individuals, four were professional climbers and 12 were engaged in regular physical activities. In addition, we tested 15 teenagers (13–14 years, seven females, eight males, body mass 43–69 kg, mean 53 kg±7 kg) with ages corresponding to the ages of the tested macaques and some of the tested chimpanzees. For all teenagers participating in this study, we obtained written consent from their parents in addition to the subjects' assent.

### RNA-seq Data

All RNA-seq data were uploaded to the Gene Expression Omnibus (http://www.ncbi.nlm.nih.gov/geo) under accession number GSE49379.

## Supporting Information

Figure S1 **Percentage of peaks affected by postmortem delay among metabolic peaks in different datasets, as well as all peaks combined (ALL).**
(PDF)

Figure S2 **Agreement between metabolite concentration profiles across the four species measured using LC-MS and GC-MS data and the independently measured CE-MS dataset.** The colored bars represent observed distribution of Pearson correlation coefficients between datasets in each tissue; the gray bars represent distributions obtained by 100 random permutations of metabolite labels. In all tissues the correlations between concentration profiles measured by different technologies is significantly higher than expected by chance (Wilcoxon test, *p*<0.001).
(PDF)

Figure S3 **PCA plots based on normalized intensities of all metabolite peaks detected in the three datasets separately.**
(PDF)

Figure S4 **Silhouette values of the clustering of the peaks according to their concentration profiles among tissues within the metabolite dataset.** The silhouette value reflects the quality of clustering favoring well-separated compact clusters. The asterisks indicate the highest silhouette values observed for 19 clusters. Among them, 13 clusters were sufficiently large, i.e., cumulatively contained more than 90% of all clustered peaks, and were used in further analysis.
(PDF)

Figure S5 **Metabolite concentration profiles within the clusters pictured in** **Figure 2a** **.** Samples are ordered along the X-axis according to tissue as indicated below the axis. Peak concentration profiles normalized by their Euclidian norm are shown with gray lines. The average concentration profile of all peaks within a cluster is traced with colored lines. The star signs show average normalized concentration levels of all cluster peaks within one tissue. The total number of metabolite peaks and number of annotated peaks within a cluster are shown above each plot. The coloring of the profiles corresponds to the coloring in Figure 2a.
(TIFF)

Figure S6 **PCA plots based on normalized expression levels of 14,875 transcripts (left) and 3,537 metabolic enzymes (right) measured using RNA-seq.** The plots show 120 tissue samples from the four species, six samples per species per tissue, selected among all samples used in metabolite measurements. Each diamond represents a sample colored according to tissue identity as indicated in the legend. The transcripts used in principle component calculation were detected in at least one tissue of one species above the 5% quintile of all detected transcripts.
(PDF)

Figure S7 **Proportions of metabolite peaks showing species-specific concentration changes among the primates in different tissues.** The phylogenetic tree above the bars shows the human (red) and chimpanzee (orange) evolutionary lineages, as well as the lineage connecting the common ancestor of humans and chimpanzees with macaques (gray). The colors within the bars represent proportions of metabolite concentration changes on the corresponding lineages.
(PDF)

Figure S8 **Proportions of metabolite concentration changes on the four evolutionary lineages.** The colors indicate proportions of peaks showing species-specific concentration changes on the human (red) and chimpanzee (orange) evolutionary lineages, as well as the lineage connecting the common ancestor of humans and chimpanzees with macaques (gray), and the lineage connecting the common ancestor of humans, chimpanzees, and macaques with mice (blue). The upper panel is based on 1,535 annotated metabolite peaks, and the lower panel on 9,155 metabolite peaks that could not be annotated.
(PDF)

Figure S9 **Proportions of metabolite concentration changes on the primate evolutionary lineages based on 2,505–5,141 metabolite peaks with no zero concentration values in all individuals of all species in a given tissue.** The colors indicate proportions of peaks showing species-specific concentration changes on the human (red) and chimpanzee (orange) evolutionary lineages, as well as the lineage connecting the common ancestor of humans and chimpanzees with macaques (gray).
(PDF)

Figure S10 **Grouping of species-specific metabolites and KEGG pathways in PFC. Columns represent metabolites showing species-specific concentration profiles in PFC; rows represent KEGG pathways enriched in the respective metabolites.** Colors show higher (red) and lower (blue) species-specific concentration levels. All pathways, including those not supported by expression of enzymes, are included in this plot. Pathways not containing enzymes with matching expression profile are indicated by lighter shades of red and blue. Metabolites directly linked to the enzymes with a human-specific expression profile in these tissues are indicated by darker shades of red and blue. Numbers and borderlines of different shades of gray indicate metabolite groups showing correlated concentration profiles across multiple pathways. The metabolite and pathway information is further listed in Table S14.
(PDF)

Figure S11 **Clustering of species-specific metabolites and KEGG pathways in skeletal muscle.** The plot is organized as in Figure S10, the metabolite groups are listed in Table S15.
(PDF)

Figure S12 **Clustering of species-specific metabolites and KEGG pathways in kidney.** The plot is organized as in Figure S10, the metabolite group is listed in Table S17.
(PDF)

Figure S13 **Numbers of metabolite peaks differentiating the control macaque monkeys from humans (human), chimpanzees (chimp.), macaque monkeys subjected to environmental condition 1 (C1), and macaque monkeys subjected to environmental condition 2 (C2) in brain, skeletal muscle, and kidney (** ***t*** **-test** ***p*** **<0.01).** Numbers of peaks showing significant concentration differences between control macaques and humans or chimpanzees were estimated using randomly chosen six human or six chimpanzee individuals, the same numbers as for C1 and C2 macaque monkeys. The error bars show 0.05 and 0.95 quintiles of the significant peak number distributions calculated by randomly sampling six human or six chimpanzee individuals 1,000 times.
(PDF)

Figure S14 **Proportions of gene expression change on the primate evolutionary lineages based on 17,913 transcripts present in all three species.** The colors indicate proportions of transcripts showing species-specific expression level changes on the human (red) and chimpanzee (orange) evolutionary lineages, as well as the lineage connecting the common ancestor of humans and chimpanzees with macaques (gray). Genes showing differences in expression level in a particular species tissue compared with all other species in the same tissue, within the upper or lower 5% quintile of the difference distribution, were classified as species-specific in a given tissue.
(PDF)

Movie S1 **Movie demonstrating the experimental setup for the measurements of muscle strength of macaque monkeys.** It contains three example takes of the pulling individuals and three example takes of the scale measuring the strength of a pull. Recordings of the scale were used to determine the maximum pulling strength of individuals.
(MOV)

Table S1 **List of tissue samples used in metabolite measurements.** Samples also used for the transcriptome measurements using RNA-seq and additional metabolite measurements using CE-MS are marked with “1” in the respective columns. Column “outliers” shows datasets where measurements in a given sample have failed as revealed by the PCA.
(XLSX)

Table S2 **List of samples used to assess effects of postmortem delay, diet, and physical activity on metabolite concentrations.** The control samples listed in this Table represent macaques kept in the same conditions as all other macaques used in main experiment, and are age-matched with “condition 1” and “condition 2” individuals. These samples were analyzed separately, as well as combined with the main sample group in the metabolite concentration analysis.
(XLSX)

Table S3 **Effect of postmortem delay on metabolite concentrations.** Numbers of metabolite peaks affected by postmortem delay across datasets and tissues.
(XLSX)

Table S4 **Proportions of metabolite variation explained by different factors.** Shown are percentages of metabolite peaks with significant proportion of concentration variation (ANOVA *p*<0.01) explained by the following factors: tissue, species, sex, age, and RNA preservation (RNA Integrity Number–RIN). The number of samples tested was limited to 85 samples for which the full annotation including RIN was known.
(XLSX)

Table S5 **Functional analysis of metabolite variation among tissues.** KEGG pathways with significant overrepresentation of metabolites in 13 metabolite peak clusters, obtained by grouping metabolite peaks according to their concentration profiles across tissues.
(XLSX)

Table S6 **RNA-seq data summary.** The Table lists total numbers of sequenced reads and proportions of reads mapped to the genome, for each sample.
(XLSX)

Table S7 **List of peaks illustrated in** **Figure 3c** **.** Table includes concentration levels of 4,698 metabolite peaks depicted in Figure 3c in the same order (from left to right) as the primate samples shown in the Figure. The columns show the concentration values followed by *t*-test *p*-Values conducted for each species in each tissue. The last column shows annotation of peaks identified in the database search.
(XLSX)

Table S8 **Numbers of peaks showing species-specific concentration changes in each dataset.** The total number of peaks in the dataset is indicated next to the dataset name, percentages of peaks in each category are shown in brackets. The rows show numbers and percentage of changes on different evolutionary lineages: the human (human) and chimpanzee (chimp.) evolutionary lineages, as well as the lineage connecting the common ancestor of humans and chimpanzees with macaques (macaq.), and the lineage connecting the common ancestor of humans, chimpanzees, and macaques with mice (mouse).
(XLSX)

Table S9 **Excess of human-specific metabolite concentration divergence at different significance cutoffs.** Shown are the ratios of metabolite peaks showing human-specific concentration changes to peaks showing species-specific changes on the other evolutionary lineages at different significance cutoffs. The cutoffs are indicated above the respective part of the Table, the *p*-Values are corrected for multiple testing. The lineages are abbreviated as: the human evolutionary lineage (human), the chimpanzee evolutionary lineage (chimp.), the lineage connecting the common ancestor of humans and chimpanzees with macaques (macaq.), and the lineage connecting the common ancestor of humans, chimpanzees, and macaques with mice (mouse). The ratios are based on metabolite peaks from all datasets.
(XLSX)

Table S10 **Numbers of metabolites with species-specific concentration profiles detected in both [+]LC-MS and [−]LC-MS datasets.** The numbers represent the percentage of overlapping metabolites among all metabolites detected in the dataset, with the ratio of human-specific to other species-specific metabolites among the overlapping ones in parentheses. *Inf* indicates no overlapping metabolites with concentration levels specific to a given species was found between the datasets.
(XLSX)

Table S11 **Numbers of KEGG pathways containing significant overrepresentation of metabolites with species-specific concentration profiles in different tissues.** The Table shows: (1) all pathways; (2) pathways that contain at least one enzyme showing species-specific expression pattern that matches the concentration profile of the pathway metabolites; (3) pathways in which metabolites and enzymes with matching species-specific concentration and expression profiles are directly linked in KEGG annotation; (4) pathways showing significant overrepresentation of enzymes with matching species-specific expression profiles either directly or indirectly linked with pathway metabolites. The lineages are abbreviated as: the human evolutionary lineage (human), the chimpanzee evolutionary lineage (chimp.), the lineage connecting the common ancestor of humans and chimpanzees with macaques (macaq.) and the lineage connecting the common ancestor of humans, chimpanzees and macaques with mice (mouse).
(XLSX)

Table S12 **KEGG pathways containing significant overrepresentation of metabolites with human-specific concentration profiles in PFC.** The Table lists: pathways names and IDs, pathway *p*-Values, numbers of pathway metabolites showing human-specific concentration profiles, numbers of enzymes showing human-specific expression, numbers of enzymes showing human-specific expression and directly linked with metabolites showing human-specific concentration profiles, as well as *p*-Values indicating probabilities of finding equal or greater numbers of enzymes or linked enzymes in metabolites sets randomly sampled 100 times.
(XLSX)

Table S13 **KEGG pathways containing significant overrepresentation of metabolites with human-specific concentration profiles in skeletal muscle.** The Table lists: pathways names and IDs, pathway *p*-Values, numbers of pathway metabolites showing human-specific concentration profiles, numbers of enzymes showing human-specific expression profiles, numbers of pairs of directly linked enzymes and metabolites with human-specific and concentration level profiles, as well as *p*-Values indicating probabilities of finding equal or greater numbers of enzymes or linked enzymes in metabolites sets randomly sampled 100 times.
(XLSX)

Table S14 **Functional modules comprised of metabolites and pathways showing human-specific changes in PFC.** The modules (metabolite groups) are listed in the order depicted in Figure S10. For each module, metabolites, pathways and enzymes are listed. The difference in concentration levels of each metabolite between humans and chimpanzees and rhesus macaques is given next to the respective metabolite. The negative sign of this difference corresponds to lower concentration levels in humans (shown in blue in Figure S10), while positive sign corresponds to higher concentration levels in humans (shown in red in Figure S10).
(XLSX)

Table S15 **Functional modules comprised of metabolites and pathways showing human-specific changes in skeletal muscle.** The modules (metabolite groups) are shown in Figure S11. Organization of the Table is analogous to Table S14.
(XLSX)

Table S16 **KEGG pathways containing significant overrepresentation of metabolites with human-specific concentration profiles in kidney.** The Table lists: pathways names and IDs, pathway *p*-Values, numbers of pathway metabolites showing human-specific concentration profiles, numbers of enzymes showing human-specific expression, numbers of enzymes showing human-specific expression profiles, numbers of pairs of directly linked enzymes and metabolites with human-specific expression and concentration level profiles, as well as *p*-Values indicating probabilities to find equal or greater numbers of enzymes or linked enzymes in metabolites sets randomly sampled 100 times.
(XLSX)

Table S17 **Functional modules comprised of metabolites and pathways showing human-specific changes in kidney.** The modules (metabolite groups) are shown in Figure S12. Organization of the Table is analogous to Table S14.
(XLSX)

Table S18 **KEGG pathways containing significant overrepresentation of metabolites with human-specific concentration profiles in brain, cerebral cortex and CBC (cerebellar cortex).** The Table lists: pathway names and IDs, pathway *p*-Values, numbers of pathway metabolites showing human-specific concentration profiles, numbers of enzymes showing human-specific expression profiles, numbers of pairs of directly linked enzymes and metabolites with human-specific and concentration level profiles, as well as *p*-Values indicating probabilities of finding equal or greater numbers of enzymes or linked enzymes in metabolites sets randomly sampled 100 times.
(XLSX)

Table S19 **KEGG pathways containing significant overrepresentation of metabolites showing concentration level changes due to environmental treatment in macaques (condition 1 and/or condition 2).** Shown are: overlap with metabolites showing human-specific concentration profiles and presence of enzymes showing human-specific expression profiles, as well as statistical significance of these observations determined by comparison to metabolites sets randomly sampled 100 times. For comparison, overlap with metabolites showing chimpanzee-specific concentration profiles is also shown. Pathways with significant enzyme expression support are marked in red.
(XLSX)

Table S20 **Muscle strength measurements in humans, chimpanzees, and rhesus macaques.** Weight and strength of an individual's pull in each testing session is indicated. Missing sessions are marked in gray, the best result of each individual is marked in green. Maximal pulling strength normalized to an individual's body weight is shown in the last column.
(XLSX)

Table S21 **Fiber-type composition of chimpanzee and human skeletal muscle.** Fibers of different types were counted in cross-sections of frozen tissue samples from nine adult chimpanzee individuals. The variation found in the chimpanzee individuals was compared with variation previously measured in humans using the same methodology [19].
(XLSX)

## Abbreviations

ANOVA: analysis of variance
AU: arbitrary units
CBC: cerebellar cortex
CE-MS: capillary electrophoresis-mass spectrometry
FDR: false discovery rate
GC-MS: gas chromatography-mass spectrometry
HMDB: Human Metabolome Database
IS: internal standards
KEGG: Kyoto Encyclopedia of Genes and Genomes
[−]LC-MS: negative mode liquid chromatography-mass spectrometry
[+]LC-MS: positive mode liquid chromatography-mass spectrometry
NICHD: National Institute of Child Health and Human Development
PCA: principal component analysis
PFC: prefrontal cortex
RNA-seq: RNA-sequencing
RT: retention time
tRNA: transfer RNA
V1: primary visual cortex
