# Supplementary figures and images for: Exceptional Evolutionary Divergence of Human Muscle and Brain Metabolomes Parallels Human Cognitive and Physical Uniqueness
Source: PLoS Biol. 2014 May 27;12(5):e1001871. doi: 10.1371/journal.pbio.1001871 (PMC4035273; doi:10.1371/journal.pbio.1001871)

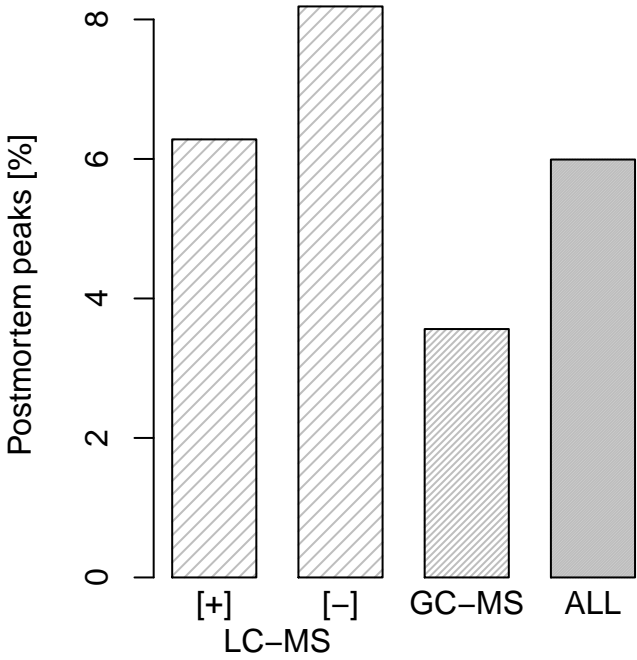

Supplement: Figure S1 — Percentage of peaks affected by postmortem delay among metabolic peaks in different datasets, as well as all peaks combined (ALL). (PDF) [file pbio.1001871.s001.pdf]

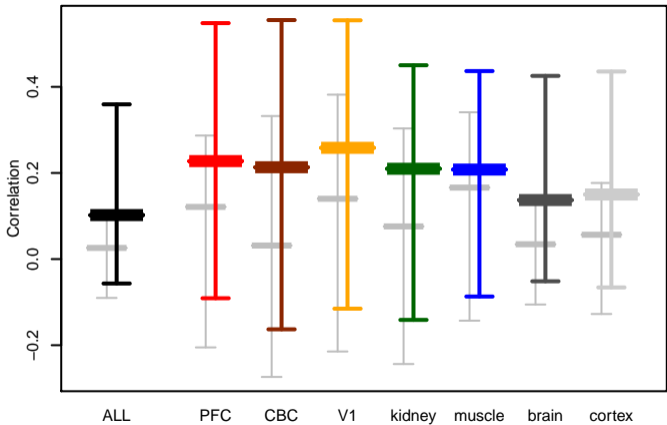

Supplement: Figure S2 — Agreement between metabolite concentration profiles across the four species measured using LC-MS and GC-MS data and the independently measured CE-MS dataset. The colored bars represent observed distribution of Pearson correlation coefficients between datasets in each tissue; the gray bars represent distributions obtained by 100 random permutations of metabolite labels. In all tissues the correlations between concentration profiles measured by different technologies is significantly higher than expected by chance (Wilcoxon test, p<0.001). (PDF) [file pbio.1001871.s002.pdf]

[+]LC-MS

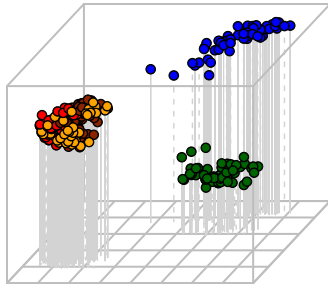

- PFC
- CBC
- V1
- kidney
- muscle

[-]LC-MS

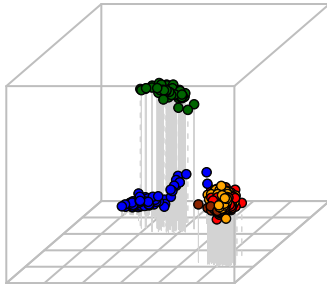

GC-MS

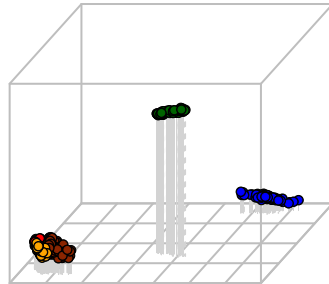

Supplement: Figure S3 — PCA plots based on normalized intensities of all metabolite peaks detected in the three datasets separately. (PDF) [file pbio.1001871.s003.pdf]

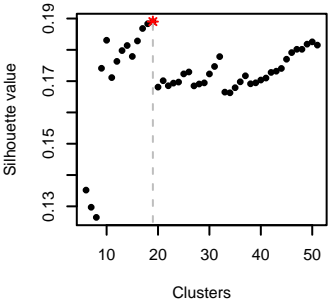

Supplement: Figure S4 — Silhouette values of the clustering of the peaks according to their concentration profiles among tissues within the metabolite dataset. The silhouette value reflects the quality of clustering favoring well-separated compact clusters. The asterisks indicate the highest silhouette values observed for 19 clusters. Among them, 13 clusters were sufficiently large, i.e., cumulatively contained more than 90% of all clustered peaks, and were used in further analysis. (PDF) [file pbio.1001871.s004.pdf]

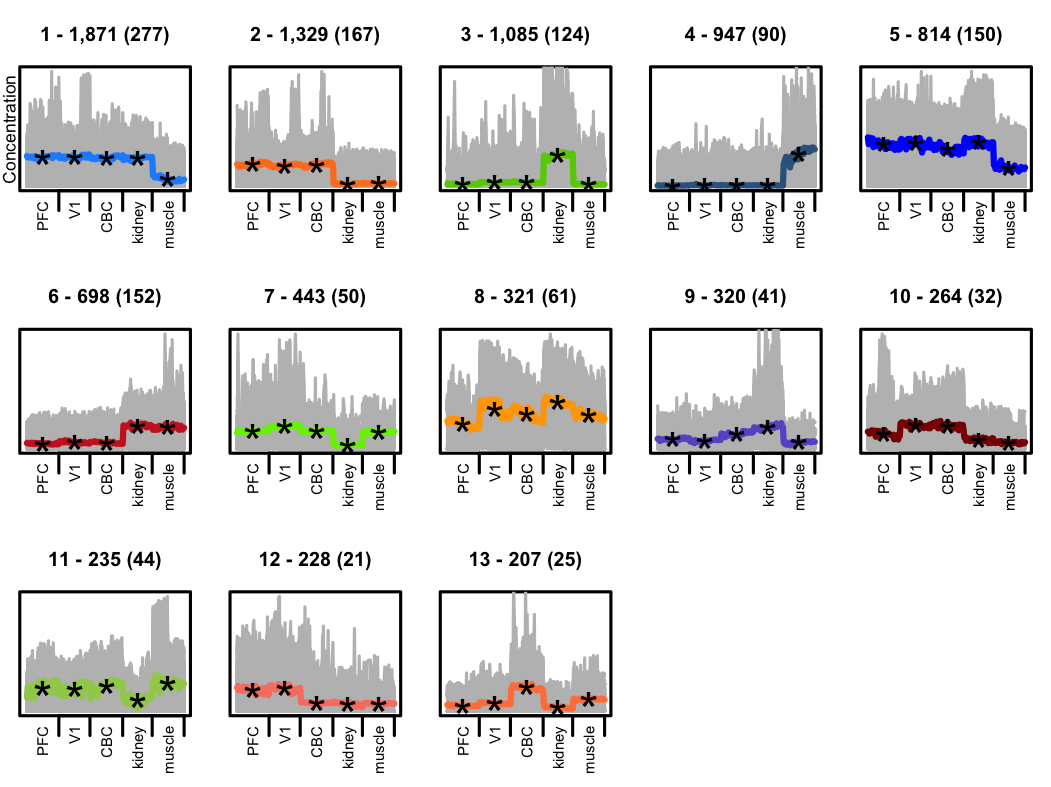

Supplement: Figure S5 — Metabolite concentration profiles within the clusters pictured in Figure 2a . Samples are ordered along the X-axis according to tissue as indicated below the axis. Peak concentration profiles normalized by their Euclidian norm are shown with gray lines. The average concentration profile of all peaks within a cluster is traced with colored lines. The star signs show average normalized concentration levels of all cluster peaks within one tissue. The total number of metabolite peaks and number of annotated peaks within a cluster are shown above each plot. The coloring of the profiles corresponds to the coloring in Figure 2a. (TIFF) [file pbio.1001871.s005.tif]

Enzyme expression

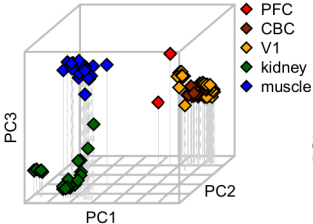

Enzymes

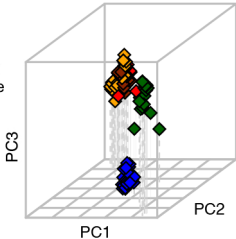

Supplement: Figure S6 — PCA plots based on normalized expression levels of 14,875 transcripts (left) and 3,537 metabolic enzymes (right) measured using RNA-seq. The plots show 120 tissue samples from the four species, six samples per species per tissue, selected among all samples used in metabolite measurements. Each diamond represents a sample colored according to tissue identity as indicated in the legend. The transcripts used in principle component calculation were detected in at least one tissue of one species above the 5% quintile of all detected transcripts. (PDF) [file pbio.1001871.s006.pdf]

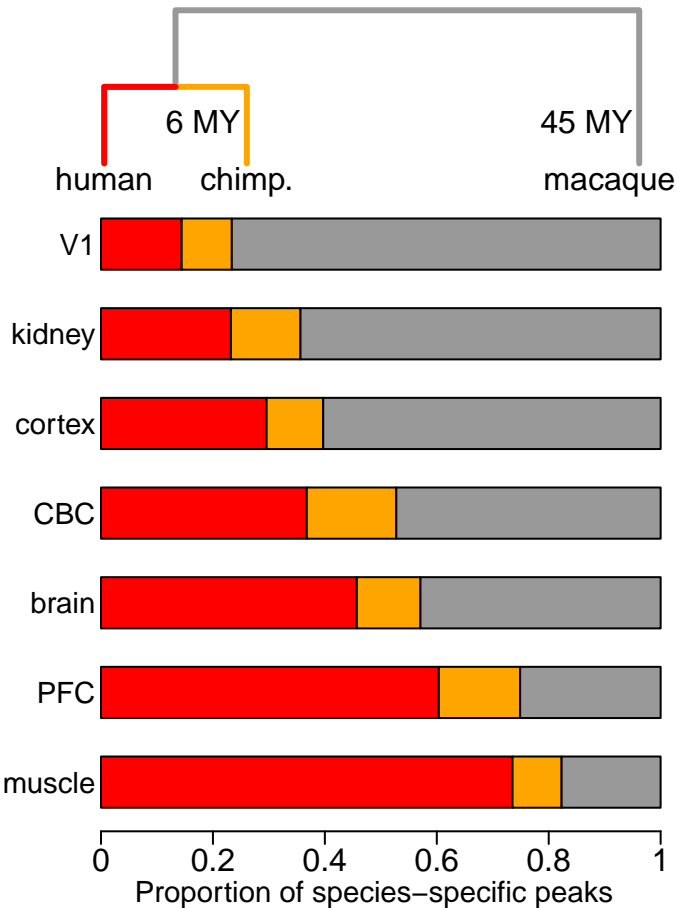

Supplement: Figure S7 — Proportions of metabolite peaks showing species-specific concentration changes among the primates in different tissues. The phylogenetic tree above the bars shows the human (red) and chimpanzee (orange) evolutionary lineages, as well as the lineage connecting the common ancestor of humans and chimpanzees with macaques (gray). The colors within the bars represent proportions of metabolite concentration changes on the corresponding lineages. (PDF) [file pbio.1001871.s007.pdf]

## Annotated peaks

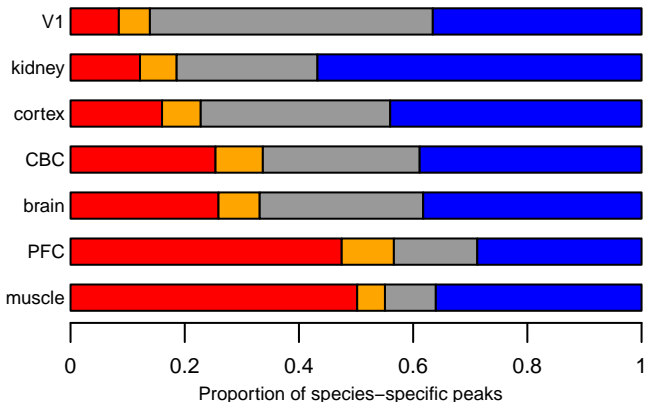

## Non-annotated peaks

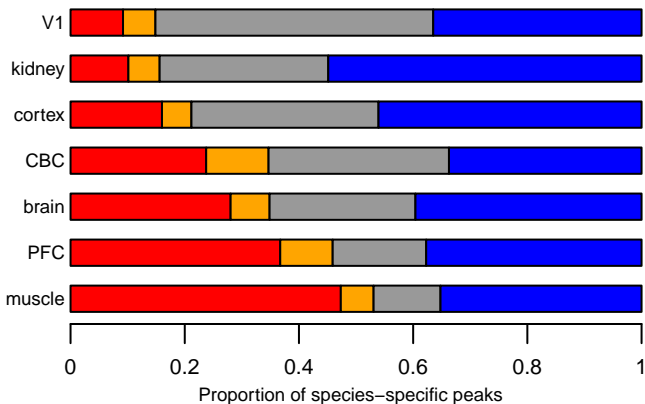

Supplement: Figure S8 — Proportions of metabolite concentration changes on the four evolutionary lineages. The colors indicate proportions of peaks showing species-specific concentration changes on the human (red) and chimpanzee (orange) evolutionary lineages, as well as the lineage connecting the common ancestor of humans and chimpanzees with macaques (gray), and the lineage connecting the common ancestor of humans, chimpanzees, and macaques with mice (blue). The upper panel is based on 1,535 annotated metabolite peaks, and the lower panel on 9,155 metabolite peaks that could not be annotated. (PDF) [file pbio.1001871.s008.pdf]

## High-concentration peaks

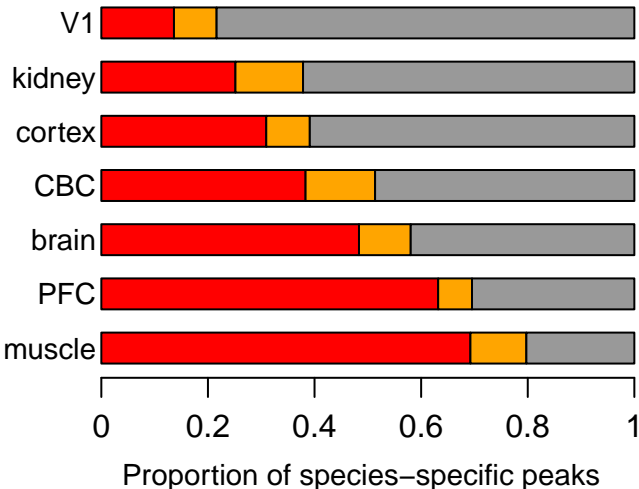

Supplement: Figure S9 — Proportions of metabolite concentration changes on the primate evolutionary lineages based on 2,505–5,141 metabolite peaks with no zero concentration values in all individuals of all species in a given tissue. The colors indicate proportions of peaks showing species-specific concentration changes on the human (red) and chimpanzee (orange) evolutionary lineages, as well as the lineage connecting the common ancestor of humans and chimpanzees with macaques (gray). (PDF) [file pbio.1001871.s009.pdf]

# PFC

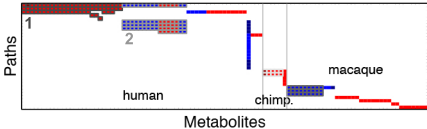

Supplement: Figure S10 — Grouping of species-specific metabolites and KEGG pathways in PFC. Columns represent metabolites showing species-specific concentration profiles in PFC; rows represent KEGG pathways enriched in the respective metabolites. Colors show higher (red) and lower (blue) species-specific concentration levels. All pathways, including those not supported by expression of enzymes, are included in this plot. Pathways not containing enzymes with matching expression profile are indicated by lighter shades of red and blue. Metabolites directly linked to the enzymes with a human-specific expression profile in these tissues are indicated by darker shades of red and blue. Numbers and borderlines of different shades of gray indicate metabolite groups showing correlated concentration profiles across multiple pathways. The metabolite and pathway information is further listed in Table S14. (PDF) [file pbio.1001871.s010.pdf]

# muscle

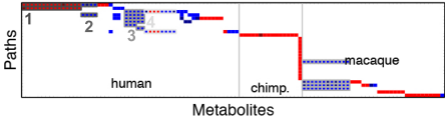

Supplement: Figure S11 — Clustering of species-specific metabolites and KEGG pathways in skeletal muscle. The plot is organized as in Figure S10, the metabolite groups are listed in Table S15. (PDF) [file pbio.1001871.s011.pdf]

# kidney

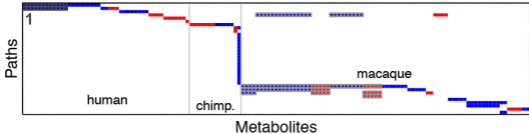

Supplement: Figure S12 — Clustering of species-specific metabolites and KEGG pathways in kidney. The plot is organized as in Figure S10, the metabolite group is listed in Table S17. (PDF) [file pbio.1001871.s012.pdf]

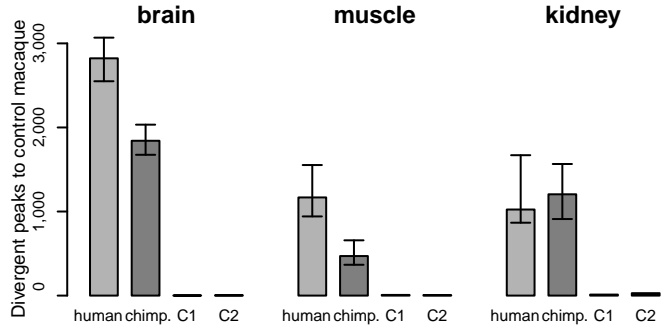

Supplement: Figure S13 — Numbers of metabolite peaks differentiating the control macaque monkeys from humans (human), chimpanzees (chimp.), macaque monkeys subjected to environmental condition 1 (C1), and macaque monkeys subjected to environmental condition 2 (C2) in brain, skeletal muscle, and kidney ( t -test p <0.01). Numbers of peaks showing significant concentration differences between control macaques and humans or chimpanzees were estimated using randomly chosen six human or six chimpanzee individuals, the same numbers as for C1 and C2 macaque monkeys. The error bars show 0.05 and 0.95 quintiles of the significant peak number distributions calculated by randomly sampling six human or six chimpanzee individuals 1,000 times. (PDF) [file pbio.1001871.s013.pdf]

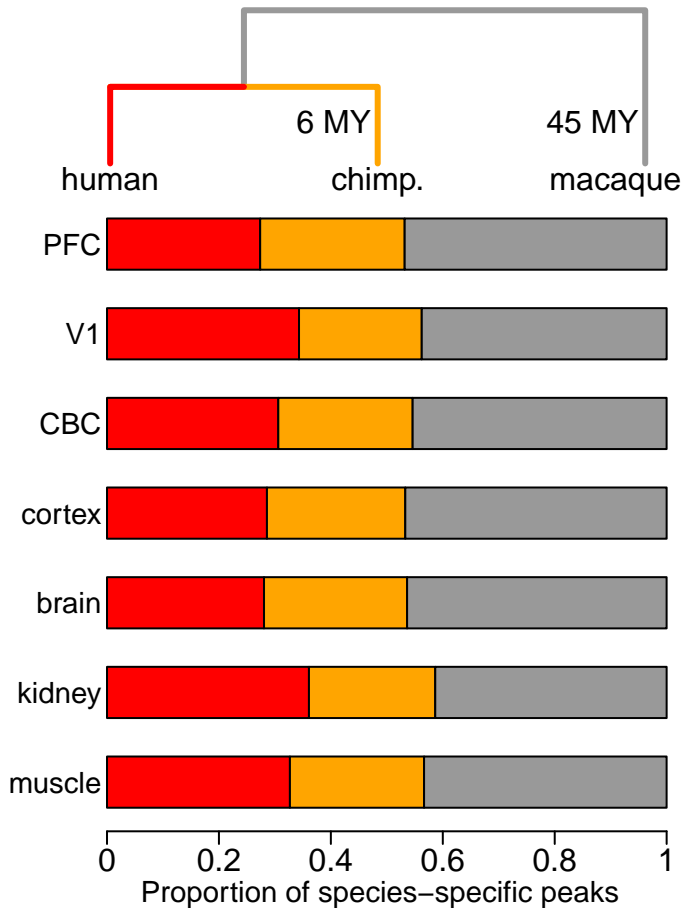

Supplement: Figure S14 — Proportions of gene expression change on the primate evolutionary lineages based on 17,913 transcripts present in all three species. The colors indicate proportions of transcripts showing species-specific expression level changes on the human (red) and chimpanzee (orange) evolutionary lineages, as well as the lineage connecting the common ancestor of humans and chimpanzees with macaques (gray). Genes showing differences in expression level in a particular species tissue compared with all other species in the same tissue, within the upper or lower 5% quintile of the difference distribution, were classified as species-specific in a given tissue. (PDF) [file pbio.1001871.s014.pdf]
